# Talking about quality: how ‘quality’ is conceptualized in nursing homes and homecare

**DOI:** 10.1186/s12913-021-06104-0

**Published:** 2021-01-30

**Authors:** Ingunn Aase, Eline Ree, Terese Johannessen, Torunn Strømme, Berit Ullebust, Elisabeth Holen-Rabbersvik, Line Hurup Thomsen, Lene Schibevaag, Hester van de Bovenkamp, Siri Wiig

**Affiliations:** 1 SHARE- Centre for Resilience in Healthcare, Faculty of Health Sciences, University of Stavanger, Stavanger, Norway; 2 Førde municipality, Førde, Norway; 3 Department of Health and Nursing Sciences, University of Agder, Kristiansand Municipality, Kristiansand, Norway; 4 Stavanger municipality, Stavanger, Norway; 5 Erasmus School of Health Policy and Management, Erasmus University, Rotterdam, the Netherlands

**Keywords:** Conceptualization, Nursing homes, Homecare; Norway, Netherlands

## Abstract

**Background:**

The delivery of high-quality service in nursing homes and homecare requires collaboration and shared understanding among managers, employees, users and policy makers from across the healthcare system. However, conceptualizing healthcare professionals’ perception of quality beyond hospital settings (e.g., its perspectives, defining attributes, quality dimensions, contextual factors, dilemmas) has rarely been done. This study therefore explores the meaning of “quality” among healthcare managers and staff in nursing homes and homecare.

**Methods:**

The study applies a cross-sectional qualitative design with focus groups and individual interviews, to capture both depth and breadth of conceptualization of quality from healthcare professionals in nursing homes and homecare. We draw our data from 65 managers and staff in nursing homes and homecare services in Norway and the Netherlands. The participants worked as managers (*n* = 40), registered nurses (RNs) or assistant nurses (*n* = 25).

**Results:**

The analysis identified the two categories and four sub-categories: “Professional issues: more than firefighting” (subcategories “professional pride” and “competence”) and “patient-centered approach: more than covering basic needs” (subcategories “dignity” and “continuity”). Quality in nursing homes and homecare is conceptualized as an ongoing process based on having the “right competence,” good cooperation across professional groups, and patient-centered care, in line with professional pride and dignity for the patients.

**Conclusion:**

Based on the understanding of quality among the healthcare professionals in our study, quality should encompass the softer dimensions of professional pride and competence, as well as a patient-centered approach to care. These dimensions should be factors in improvement activities and in daily practice.

**Supplementary Information:**

The online version contains supplementary material available at 10.1186/s12913-021-06104-0.

## Background

Quality in healthcare is a multidimensional concept that incorporates clinical effectiveness, patient safety, and patient experiences [1]. In this article we investigate the ways in which healthcare professionals and staff in Norway and the Netherlands conceptualize quality in nursing homes and homecare. We elaborate on previous research on conceptualizations of quality in healthcare, describe the knowledge gaps, and present the rationale for the study.

### Previous research

Previous research shows that the delivery of high-quality healthcare requires collaboration and a shared understanding of quality among policy makers, managers, employees, and users [2]. However, the conceptualization of quality depends on the local context, and may vary across the macro, meso and micro levels of the healthcare system and among different stakeholders, such as nurses, doctors, and managers [3–6]. From research in hospital settings, we know that managers and staff understand quality in different ways [5]. Managers, for example, prioritize quality indicators and performance. Healthcare professionals, in contrast, focus on clinical effectiveness and patient centeredness. There are also different emphases based on professional roles, personal ideas and beliefs [5].

There is a growing knowledge about conceptualization of quality in the hospital setting (e.g. [5],), but there is less research on the topic from the nursing homes and home care settings However, a notable exception, the UK-based study by Farr and Cassey [7], concluded that nursing homes and homecare staff’s notion of quality was influenced by their personal and professional values and standards.

At the organizational level, quality and performance in healthcare tend to be rooted in different organizational logics and may appear paradoxical, because of the tensions between patient-centered and relational care, on one hand, and the pressures of efficiency and rationalization on the other [5, 7–10]. A recent study illuminates several organizational variables (e.g., plural consensus, distributed connectedness, patient coreness) that need to be balanced to control quality improvements activities [8]. Moreover, person-centered care is becoming increasingly important in the discussion of healthcare quality. Some researchers have questioned whether rule-based quality systems can be aligned with and adapted to person-centered care, which emphasizes the patient perspective [6, 8–10]. Other studies point to shortages of staff and resources as key factors that limit both the conceptualization and provision of high-quality service [11–14].

The many meanings of quality among healthcare professionals, managers and regulators indicate a need to confront quality paradoxes and identify new characteristics of “quality” in contemporary healthcare [1, 6–8, 14–16]. Should, for example, standards of care quality be more deeply rooted in the lived experience of patients, employees and managers? [15, 17].

The multifaceted notion of quality poses a challenge to managers and staff who implement quality systems and ensure safe operations, suggesting that the concept of quality should be thoroughly understood and grounded in qualitative studies [5, 8]. This has led to an ongoing and changing dialogue of what quality means in nursing homes and homecare that constitutes the key motivation and focus of our study, where we examine this phenomenon in two countries – Norway and the Netherlands.

### Quality definitions

There are many definitions of quality, many of which mention effectiveness, timeliness, safety, equity, efficiency and patient-centeredness [18–20]. In Norway, the public definition of quality in healthcare is based on meeting society’s demands, complying with legislative requirements, and providing users with sound professional practice. For health and social services, “high quality” means that services are effective, safe and secure, involve and empower users, are coordinated and continuous, utilize resources efficiently and are available and evenly distributed [21, 22].

In the Netherlands, the public definition of good quality care is “at least safe, effective, efficient and client centered, is provided timely and is attuned to the real needs of the client” [23]. In addition, good quality care means that care professionals act according to their professional responsibility and standards. Moreover, good quality care takes into account the rights of clients and treats clients with respect [23].

We need to improve our understanding of the quality challenges in different countries [5] and identify key quality dimensions as understood by healthcare professionals in nursing homes and homecare. This is potentially important, for example, when targeting interventions and setting strategic quality priorities.

### The SAFE-LEAD nursing home and homecare study

This paper reports from the project “Improving Quality and Safety in Primary Care – Implementing a Leadership Intervention in Nursing Homes and Homecare” (SAFE-LEAD) [24] exploring quality and safety work in nursing homes and homecare in Norway and the Netherlands (see Wiig et al. [24] for the full study protocol). The aim of the SAFE-LEAD project is to build leadership competence and guide nursing home and homecare managers in advancing and improving vital quality and safety strategies, attitudes and practices in their organizations [24, 25]. Healthcare professionals’ understanding of quality is an important contribution to this aim. By focusing on healthcare professionals’ conceptualization of quality in Norway and the Netherlands, this paper contributes to closing the knowledge gaps in the nursing home and homecare settings.

### Aim and research question

The aim of this study is to explore the meaning of quality among healthcare managers and staff in nursing home and homecare settings and to discuss any differences and similarities among these professionals. This paper addresses the following research question: How do healthcare managers and staff conceptualize quality in nursing homes and homecare settings?

By identifying how healthcare managers and staff in Norway and the Netherlands understand quality, the study generates new knowledge from these healthcare professionals and supports a shared understanding of quality as the cornerstone of future quality improvement efforts.

## Methods

In this section, we introduce the study contexts in Norway and the Netherlands [26]. We then describe the study design, sample, data collection and data analysis.

### Context

The Norwegian healthcare system is semi-decentralized, meaning that the parliament is the national decision-making body. The municipalities are responsible for primary health care, which includes homecare and nursing homes. The municipalities organize nursing home and homecare services, and there is no direct command and control line from national authorities to the municipalities [27]. The central government focuses on patients’ health and care service; placing their needs at the center of healthcare provision [28], but there is no dedicated law for the nursing home and homecare users. Norway’s Patient and User Rights Act [29] is comprehensive legislation to ensure high quality of care and safety of services. Moreover, national professional guidelines enacted and enforced by the Directorate of Health are designed to ensure a high quality of care [30].

From 2010 until 2018, a National Patient Safety Program improved quality and safety by strengthening the competence of healthcare staff and managers and implemented measures to reduce harm to patients. Participation was mandatory for the hospitals, but voluntary for municipalities [31]. A new national action plan (2019–2023) for patient safety and quality improvement emphasizes the need for structures, cultures, and competence to reduce patient harm in both specialized and nursing home and homecare services. A main challenge is the lack of knowledge about patient safety incidents and harm in nursing home and homecare [18].

The Dutch healthcare system is a hybrid system. Homecare and nursing home care are governed at the national and local (municipal) levels. The Dutch governance system combines top-down government regulation, market elements (free choice of provider and insurer), and self-regulation by professionals and consultation amongst relevant actors [32]. Since the 1980s, improvements in quality and safety and strengthening the position of patients have figured on the agenda [26].

Healthcare and elderly care in the Netherlands are regulated and financed through a variety of acts. The Health Insurance Act [33] regulates acute and curative care through private insurance (and some small service out-of-pocket payments). The Long-term Care Act [34] is paid for through taxes and for some service small out-of-pocket payments. The Social Support Act [35] also works through a combination of taxes and out-of-pocket payments.

Several national quality programs have focused on elderly care, for example, emphasizing patient-centered care, regional network building and the reduction of regulatory pressure [26].

### Design, setting and sample

The study applies a cross-sectional qualitative design [36] using both focus groups and individual interviews to capture the depth and breadth of conceptualizations of quality from healthcare professionals working in nursing homes and homecare.

The main study setting is nursing homes and homecare services in Norway. A smaller study of healthcare professionals with different roles in the Dutch elderly services was also conducted. Through purposeful sampling the units were strategically selected to represent urban and rural) locations in addition to municipalities and units of different sizes.

We include data from 13 municipalities: nine in Norway and four in the Netherlands. Seventeen units were included; ten nursing homes and seven homecare services. Sixty-five healthcare professionals participated. They were registered nurses (RN) or assistant nurses (*n* = 25), and managers (*n* = 40). The number of managers (*n* = 40) include both managers at nursing homes and homecare (*n* = 33), quality managers (*n* = 5), and directors of health and welfare services at the municipal level (*n* = 2) (Table 1). The quality managers worked as quality advisors and consultants for both nursing homes and home care services in the Netherlands. Quality managers in Netherlands can work across municipalities and units within nursing homes and homecare services.

**Table 1:** Data Sampling and Setting

		(estimated N of inhabitants)	Units (n)	Staff (n)	Managers (n)	Total (n)
M1	Norway	15–20,000	1 HC (homecare)	6 (HCS)	4 (HC)	10
M2	Norway	70–75,000	1 HC 2 NH (Nursing home)	5 (NH)	1 (HC) 2 (NH) 1 (Director of healthand welfare services)	9
M3	Norway	< 5000	1 HC	0	2 (HC) 2 (NH)	4
M4	Norway	5–10,000	2 NH	4 (HCS) 4 (NH)	3 (HC) 3 (NH)	14
M5	Norway	< 5000	Municipality level	0	1 (Director of healthand welfare services)	1
M6	Norway	10–15,000	1 HC	0	2 (HC)	2
M7	Norway	130–135,000	1 NH	6 (NH)	9 (NH)	15
M8	Norway	15–20,000	1 NH	0	1 (NH)	1
M9	Norway	20–25,000	1 HC 2 NH	0	1 (HC) 2 (NH)	3
M 10	Netherlands		1 HC	0	1	1
M11	Netherlands		1 NH	0	3 Quality Managers	3
M12	Netherlands		1HC	O	1 Quality manager	1
M13	Netherlands		1NH	0	1 Quality manager	1
**Total**			17 (10NH, 7 HC)	25	40	**65**

The COREQ Checklist is filled in (see Additional file 4).

There are more participants in the Norwegian sample (*n* = 59) than in the Dutch sample (*n* = 6), because the main component in the SAFE-LEAD project relates only to the Norwegian context. Both the municipalities and the units varied in size. The project leader (SW) in the SAFE-LEAD contacted the unit manager in each unit with a request to recruit contact persons within the units. Participants from each unit included both managers and staff. Each unit selected its participants for our study. No one who was recruited to participate declined.

### Data collection

Data collection methods consisted of individual and focus group interviews with healthcare managers and staff. All researchers (IA, TJ, TS, BU, EHR, LHT, HB, LS and SW) in the.

SAFE-LEAD project participated in data collection and conducted all individual and focus group interviews. Two researchers (a moderator and an observer/secretary) conducted the focus group interviews; that lasted some 90 min. One researcher conducted the individual interviews that lasted approximately 45 min. One researcher (TJ) from the SAFE-LEAD project conducted the interviews with the Dutch participants. The Dutch researcher (HB) organized the recruitment and data collection in the Netherlands. Seven of the researchers (IA, TJ, TS, BU, EHR, LHT and LS) have clinical experiences and all have participated at some point in the SAFE-LEAD implementation. The research team has a multidisciplinary background that includes nursing, safety science, health science, and health psychology. All have been trained and have expertise in qualitative methods. All data collection was performed at the participants’ workplace during their regular working hours. The data collection was conducted from May 2017 to April 2018 according to semi-structured interview guides based on the “Organizing for Quality Framework” (OQ) [37], adapted to the Norwegian nursing home and homecare settings [25]. The interview guides included topics related to several quality challenges: structure, culture, engagement, competence, care coordination and organizational politics, external demands, and physical design and technology (see Additional files 1, 2 and 3). The interview guides varied between healthcare managers and the staff and there were some differences in the questions about competence development; the managers explained how they facilitated competence development, while the staff described whether and how they got time and possibilities to enhance their competence. Nevertheless, the most important question — how they understood “quality in primary healthcare”— remained the same in all interviews.

Data collection consisted of 15 individual interviews, six focus group interviews with managers, and eight focus group interviews with staff in nursing homes and homecare. Malterud et al. [38] suggest that the concept of *information power* should be used to guide adequate sample size for qualitative studies. We assessed that the sample had sufficient information power to conduct a sound analysis. According to Malterud et al. [38], having a specific research question and a relevant sample to explore it, using a theoretical framework as guidance, having high quality of the dialogue, and an in-depth case analysis strategy increase information power. We collected data until we reached saturation, implying that all perspectives had been covered and elaborated. All the data from both the individual interviews and the focus group interviews were audio recorded and transcribed verbatim.

### Data analysis

Researchers in the SAFE-LEAD project conducted and analyzed both the Norwegian and Dutch interviews. We analyzed all of the data material according to conventional content analysis [39], to describe managers’ and staff’s conceptualization of quality in nursing homes and homecare. Conventional content analysis is an inductive methodology in which coding categories are derived directly from text data, and the researcher avoids using predefined categories. This methodology was considered appropriate, given the explanatory and open nature of our research question, asking how healthcare professionals understand and conceptualize quality in healthcare. Although the interview guides were based on the organizing for quality framework [37], they were semi-structured, allowing an inductive analysis to be conducted. The categories in the framework were used as guidance to cover relevant aspects of quality in healthcare, while our analysis is based on the study research question.

All the transcripts from both focus group and individual interviews were handled manually and systematized in tables in a Word document without the use of any software for qualitative analysis.

The research team-members: IA, ER, TJ, TS, BU, EHR, LHT, LS, HB and SW contributed to the data analysis which started by identifying and initially coding data in the material about the respondents’ “understanding of quality.” The researchers IA, ER, TJ, TS, BU, EHR, LHT, LS, and SW read and then reread the transcribed material to get a perception of the quality aspects mentioned by the healthcare staff and managers and to find nuances and breadth in the data.

Two authors (IA/SW) derived codes that captured essential meanings and thoughts related to the research question. Codes with similar meanings were then grouped into categories and subcategories. To ensure trustworthiness in the analysis, the preliminary analysis and emerging categories were discussed face-to-face in several iterations of researcher meetings and SAFE-LEAD project meetings with all participants (IA, ER, TJ, TS, BU, EHR, LHT, LS, HB and SW). IA prepared and organized four face-to-face meetings with the research team, and two meetings with the whole SAFE-LEAD project group including the co-researchers. These meetings ensured that the researchers could recognize the results categories and content. After everyone agreed, we finalized the categories, subcategories and codes.

## Results

The analysis identified two categories and four sub-categories that resonated across all participants, both managers and staff in individual and focus group interviews. The categories are labeled “professional issues: more than firefighting” (subcategories professional pride and competence) and “patient-centered approach: more than covering basic needs” (subcategories dignity and continuity).

When analyzing data, the categories for managers and staff were similar, but with some variations in the content of the sub-categories and codes. For example, when managers talked about competence they talked about deviation, reporting system, and education. In contrast, the staff talked about updated and regular information about new procedures, patients and organizational issues, time, equipment and training opportunities. There was general agreement among our participants, though we have included some quotes to show that there are slightly different views and divergences between Norway and the Netherlands.

The following sections present the two categories, with their corresponding sub-categories and codes (Table 2).

**Table 2:** Categories, Sub-Categories and Codes

Categories	Sub-Categories	Codes
Professional issues: more than putting out fires	Professional pride	Responsibility Ethical reflection Being professional
	Professional competence	Training Information Deviation and indicators
Patient-centered approach: more than covering basic needs	Continuity	Cooperation Common understanding Common tools
	Dignity	Patient first Personalized care Secure and good care

### Professional issues: more than putting out fires

This study shows that the participants conceptualized quality as more than putting out fires, focusing on ongoing processes related to professional pride and competence. They illuminated that poor quality was often associated with a sense of being under pressure from external demands and cited several times when they had acted on these demands to remain professional and to offer the best quality of care to the patients, for instance by making time for them to have their hair and nails done.

### Professional pride

The participants identified professional pride as an important dimension of quality in primary healthcare. The nurses were proud of their ability to offer high-quality care to each patient. They described quality as good when they took responsibility for their work and had time to exchange ethical reflections with their colleagues. These reflections were, for example, related to patient care, cooperation with next of kin and the resolution of dilemmas about scarce resources and insufficient time for each patient. The staff reported that professional pride was important for providing high-quality care and equated with being a good “fellow human.” The managers cited professional pride as giving care of highest quality, professionalism and having enough staff to attend to patients. The motivation of managers and staff resulted from providing patients with the extra service. This was at the heart of their conceptualization of “quality work.” In the words of one manager:

“Quality is not just firefighting, it is when we do this extra for the patient, then I am proud of my work” (Manager, Norwegian NH, M4).

Managers talked about being a good motivator for the staff, to have enough staff at work, and manage periods with sick leave as prerequisite for providing care quality to their patients. They also highlighted the importance of spending more time with and being visible to their staff. Managers argued the patients would benefit from better care as a result of this leadership approach. Moreover, they argued that checklists and reporting on indicators were mandatory activities, often without added value for the patient, hence it did not contribute to a sense of professional pride. Some talked about the benefit of not using time on completing checklists, because they preferred face-to-face time with the patients. One participant from the Netherlands commented that checklist registration is different from what staff are doing, because:

“the people who are working daily with the elderly people they do not always think about quality, but they are nevertheless doing quality work” (Quality manager, Dutch NH, M13).

Findings also indicate that when participants see the function of the checklist or indicator, they see the added value (e.g., medication safety). Other participants noted that quality of care improved when the staff customized tasks to their patients’ preferences.

### Professional competence

Conceptualization of quality was closely related to professional competence. Both managers and staff talked about the importance of having the competence for the provision of quality care. Some managers talked about the challenges of sharing knowledge within the unit because there were so many part-time employees. The staff had a constant need for updating, learning and training on new procedures due to new and advanced treatment options for the patients treated in the municipalities.

Two examples are dialysis and immunotherapy. A few years ago, in Norway, only hospitals provided these treatments; today these are provided to patients at home or in nursing homes. These tasks require high nursing competence, the resources to use certain procedures, and compliance with procedures and regulation. Competence as a quality dimension, especially among managers, was linked to having the right professionals in the right place at the right time. There also had to be enough nurses to attend to patients with special needs. Moreover, the managers highlighted that staff needed to be empowered, independent, and “self-propelled.” According to a nursing home manager:

Quality of care depends on regulations being followed, and that we can trust the staff to follow the rules and procedures. (Manager, Norwegian NH, M2)

At the same time, the staff insisted that high quality of care was depended on them receiving sufficient training to meet requirements and understand new forms for treatment. In addition, the staff often needed more information to do a better job and to improve service quality.

Both managers and staff emphasized the need for higher competence in medication administration, check-list registration, documentation and nursing procedures such as nutrition guidelines. The staff was trained on new procedures, documentation systems, as well as the error reporting system to build competence. They cited the importance of training to be confident and to enjoy their work. It seemed important for the staff to trust their competence. This included updated and regular information about new procedures, patients and organizational issues. The staff asked for courses to maintain and develop their competence. Most of the participants talked about the necessity to report deviations not only regarding serious events but also near misses to improve quality in healthcare.

Participants from the Netherlands had several quality indicators, but this was less common among the Norwegian sample. All participants emphasized the importance of procedures with documentation and registration but complying with procedures and registration was not enough to ensure quality of care. One manager said*:*

“Quality cannot be measured on a tablet, it is not the basic functions and measurement that represent quality for a patient, but it is the dignity and empowerment that make the difference,” (Manager, Norwegian NH, M7)

The Dutch participants also mentioned the importance of staff having a sense of ownership of their work. They stated that each healthcare professional must know what to do and why in order to offer the best care to their patients.

### Patient-centered approach: more than covering basic needs

The category patient-centered approach includes the sub-categories continuity and dignity.

### Continuity

The participants conceptualized quality in relation to the predictability and continuity of care. Continuity of care by ensuring a low staff-to-patient ratio was important, both for themselves and for the patients. Continuity of care contributed to their feeling of doing a better job for the patients.

Cooperation emerged from the data as an important dimension in the participants’ conceptualization of quality. All participants mentioned the importance of a collaborative workplace, where it was physically and socially easy to obtain help and support. They talked about cooperation intra-professionally within the nursing unit, and inter-professionally with physicians, physiotherapists, social workers, professionals, and politicians.

From the managers’ point of view, the quality of care depended on cooperation among professionals, exchange of experiences and professional support within and across units. For example, managers advocated for teamwork as essential in the “everyday rehabilitation” of patients. The quality of a patient’s rehabilitation was based on teamwork, because patients need to be as physically active as possible, implying the need for a shared understanding of sound care within the team, and a good dialogue between the professionals and the patients. Both managers and staff wanted to see more cooperation with the physiotherapist and the occupational therapist. As one manager said:

“I am not satisfied (happy) with the fact that physio and occupational therapists do not work shifts. That leads to poor quality.” (Manager, Norwegian HC, M9)

The healthcare staff supported each other and sometimes covered each other’s tasks at the busiest times. The cooperation made them feel more secure in their work, and they argued that it contributed to improving the quality of care. In the Netherlands, interprofessional teams in which each profession contributed supplementary knowledge and perspectives was highlighted as important for quality of care. Both managers and staff reported that interprofessional communication was associated with better quality. This was also evident in the Norwegian sample. In the words of a homecare manager:

… a culture to share experiences and learn from each other, we talk a lot about it, how can you learn from each other. What in the world are some doing since they manage to get patients on their feet, while others cannot? (Manager, Norwegian HC, M2).

Conceptualization of quality was also linked to cooperation in terms of common tools for documentation. The soundness of quality depended on the ability to use documentation systems between care levels and to secure medical records and discharge summaries at the time of transfer. All participants talked about incomplete or inaccurate medication lists, often resulting in the wrong pills and medication being dispensed to the patients. They asked for more reliable communication and cooperation. Some participants cited the usefulness of e-health and “mobile care,” which gave immediate answers to their questions.

### Dignity

Dignity was an important category in the conceptualization of care quality. The participants insisted that the quality of care depended on patients being treated with dignity. They stated that good quality care, or “personalized care,” puts patient first.:

“The patients should receive services with good and sufficient quality, and this should be provided with dignity.” (Staff, Norwegian NH, M2)

The staff articulated the need for giving the patients valuable treatment and care that protected patients’ dignity. In other words, patients should be able to choose their activities, nutrition and caregivers. Dignity also pertained to their intention of enabling patients to live meaningful lives. At the same time, healthcare staff were mindful of what is most clinically effective for their patients, which sometimes created professional dilemmas. For example, some patients preferred to stay in bed even though they would benefit from physical activity. Some managers in both countries talked about quality as something that was more than measurable or tangible:

It is not basic functions and measurements that represent quality for a patient in a long-term institution. To carry out one’s life, i.e. the end of life, with a dignity and a co-determination that makes you feel alive until the end. It’s not about your blood sugar or talking to a doctor or annual checkups and that kind of things. (Manager, Norwegian NH, M4).

In our organization, we think that patients’ well-being should be prioritized above all. This discussion is ongoing - over and over again, which risks can we take for the patients and their well-being [what is the consequence]. (Quality Manager Dutch HN, M11)

The participants also conceptualized quality in terms of the provision of “best practice,” meaning that they constantly needed to make many small changes to provide optimal care, because best practices change with time. Another way to put “the patients in the center” was to sit with them while they are and to keep their rooms tidy and welcoming.

We are really serious about putting the client in the center - we have to do this. We must do this. Otherwise we have to say the client has lost. (Quality Manager Dutch HC, M12)

## Discussion

This section summarizes the key findings and comparisons with the research and literature. We use the Organization of Quality framework [37] as our theoretical lens.

The results show that conceptualization of quality in nursing homes and homecare entails more than doing the “checklists,” having the necessary competence, and collaborating within and across units and levels. Professional pride and treating the patients with dignity emerged from our study as fundamental quality dimensions as seen from perspectives of managers and staff. Quality of care in the nursing homes and homecare settings is understood as more than putting out fires, it is by having the “right competence,” cooperating, and ensuring continuity and predictability for patients who are being served by healthcare providers.

### The importance of “soft dimensions” in conceptualization of quality

Comparing our results with quality dimensions appearing in definitions by IOM [20], we see that continuity, safety, timeliness, and patient-centered care are similar to elements described in our study. However, some interesting differences need some reflection. Since “quality in healthcare” has become a powerful driver of health policy with a set of measurable indicators in several countries, it is important to illuminate the complexity of this concept which includes several softer dimensions (dignity, professional pride), identified in our study. This is consistent with previous research in hospitals [5, 8], nursing homes and homecare [7]. Among the quality challenges that service providers need to address to improve the quality of care [24, 36], our findings fit most with the soft dimensions: “engagement,” “competence” and “culture” [25, 37, 40] in the Organizing for Quality framework. The challenges regarding “engagement” or “enthusiasm” [25, 37] are similar to our result regarding the subcategories of professional pride (responsibility, ethical consideration and being professional) and of dignity (personalized care, patient first and secure and good care). The cultural and competence challenges in the framework, centered on building common values, knowledge, and learning [25, 37], have similar content as the subcategories; professional competence and continuity and their codes in our study (e.g., cooperation, common understanding and training).

These soft dimensions of quality, supported in earlier research [5, 7, 8], cannot be easily measured, but they are important for healthcare managers’ and staff’s experience of providing quality care and needs to be reflected in healthcare systems effort to improve the quality of service in nursing homes and homecare. This relates to the study by Bouwman et al. [6] who found a disparity between the perspectives of patients and regulators on healthcare quality, and with Nunes et al. [8] who advocated for a move from project-based and systematic thinking about quality improvement to balancing the simultaneous working on opposing ideas. The softer dimensions may also be in line with patient and family perspectives on quality in nursing homes and homecare; Bouwman et al. [6] found that patients seem to tolerate clinical errors more than relational deficiencies in care provision. Managers need to understand what healthcare staff appreciate and consider important for doing a good job and to ensure quality of their services [8]. Our findings have some similarities with human resource management where, for example, Dieleman et al. [41] identify increased knowledge and skills, and health workers’ motivation as essential in human resource management. Motivations for health workers were their awareness of local problems and staff empowerment, their acceptance of new information and the creation of a sense of belonging and respect [41]. We found that healthcare staff are interested in going the extra mile for their patients, which includes protecting patients’ dignity and safeguarding their own professional pride, but in line with Gallagher et al. [42] they need sufficient support and resources to operationalize dignity in their everyday practice.

In their editorial, Swinglehurst et al. [15] argue in favor of rethinking quality in health care, stating that “every act of health care is an opportunity for unique tailoring rather than a ‘one-size-fits all’ prescription and it is this response to the complexity of each situation that marks out high quality care” (p.5). This is supported by our findings about the importance of providing each patient with continuity of care, dignity and personalized care. Also, in line with our findings, Vincent and Amalberti [2] highlight the cumulative effects of performing intangible services for the patients, such as treating them with dignity and respect, as important for quality. At the same time, our results differ from those of Fatima et al. [43], whose review found that service quality in healthcare was most often measured by technical and functional dimensions focusing on the tangible. Taking our results into account, a strong attention to measurable quality without acknowledging the importance of the intangible will most likely create tensions and dilemmas for healthcare staff and managers in the course of performing ordinary care [9].

### Acknowledging differences in quality conceptualization

Wiig et al. [5], comparing the conceptualization of quality among the macro (policy maker), meso (manager) and micro (staff) levels and countries in hospital settings, found that the conceptualization at the micro level differed from those at the meso and macro levels. Consistent with our findings, nurses at the micro level often linked high-quality care to softer dimensions of quality such as patient-centered care, talking with the patients, and the physical environment [5]. Managers, however, seemed more preoccupied than staff with external factors, indicators, and measurements. Although the managers in our study were more likely than staff to emphasize the importance of checklists and indicators, both groups seemed to value time to provide the little “extra.” However, for managers, this implied making the time to encourage and interact with their staff; for the staff this meant having time to talk with and provide high-quality care to their patients. These aspects were part of the professional pride that both managers and staff mentioned in their conceptualization of quality in nursing homes and homecare in our study, as well as in hospitals [5].

Thus, as in the study by Wiig et al. [5], our study shows that the main dimensions emphasized by managers and staff in their conceptualization of quality are similar, but there are differences in the way in which they talk about and act on these dimensions. Findings from our study echo Kelly et al.’s [44] interdisciplinary study “Unmasking quality: exploring meanings of health by doing art.” They revealed how quality in healthcare has many potential meanings and interpretations. They explored and challenged the dominant reductionist meanings of quality and argued that conceptualization must place more emphasis on describing quality and less on measuring it through structural-oriented metrics. It is essential to acknowledge and generate new knowledge about the differences in these quality conceptualizations of as a foundation for improvement work in nursing homes and homecare settings.

### Is there a need for a broader quality conceptualization?

In our result the subcategories “continuity” and “dignity” showed the importance of supporting patients and allowing them to choose their activities, nutrition and the professionals caring for them. In line with our findings, Aase et al. [45] argue that the continuity dimension of quality needs to reflect professional and good nursing care. Furthermore, it should include ethical and relational considerations, the time dimension, observations and organization, and meet the challenges of virtual follow-up and documentation across system levels [45]. In this way, continuity can create spaces and opportunities in meeting patients and their relatives, where the nurse exercises comprehensive care in a health service with increased demands for efficiency, technology and economy [46, 47].

The conceptualization of quality in healthcare in our study emphasizes the importance of understanding people’s experience of quality to succeed in quality improvement interventions. Based on our results, we argue there is a need for a more holistic understanding of quality, which integrates both the structural (well-functioning IT systems, documentations, indicators, and responsibilities), and the softer dimensions (professional pride, dignity) of quality to cover what healthcare professionals include in their meaning of delivering services of high quality. A recent scoping review of the development of a framework to describe patient and family harm, highlights a wide range of components of experiences of disrespect which may influence the quality of healthcare [48]. This framework may help to prevent non-physical harm and protect the dignity and continuity of care.

Both Norway and the Netherlands have national quality indicators, which the participants need to register as part of their work. In Norway there are fewer quality indicators related to nursing homes and homecare settings, than in specialist healthcare. The Dutch healthcare system has developed numerous indicators in healthcare, and the data in our study shows that those indicators are more prominent in the minds of the Dutch than of the Norwegian participants. This is likely because of the larger amount of indicator use, but at the same time, the Dutch participants stressed patient-centered care [14]. If healthcare professionals are familiar with indicators and if those indicators appear meaningful, they may be considered an important part of their conceptualization of quality of care.

### Limitations

This study has several limitations that must be taken into account. First, several researchers contributed to the data collection and the analysis. This can be both a strength and a limitation, but we ensured trustworthiness by organizing analysis meetings and discussions among the researchers during the analysis of the results.

Second, the sample consisted of registered nurses and nursing assistants; no physicians or patients were included. We know from previous research that nurses and physicians have different perspectives of quality [5], and we suggest conducting further studies to explore other healthcare professionals’ and patients’ conceptualization of quality in nursing homes and homecare.

Third, the Dutch sample in our study had fewer participants than the Norwegian study, which adds bias to the sample. However, the Dutch results were not used for comparative purposes, but rather to add breadth and insight to the results.

Fourth, given the qualitative approach and the purposeful sample in this study the results may have limited generalizability. However, the diversity of the nine Norwegian municipalities in the sample paint a clear picture of Norway’s municipal context. Furthermore, we have described the context and settings so that other researchers can determine whether or not the results are transferable to other healthcare contexts and countries.

## Conclusion

In this study, we have explored how staff and managers in nursing homes and homecare conceptualize quality. Quality in these settings is understood by healthcare professionals as being both proud of and having dignity in their professional work. Quality is conceptualized as an ongoing process that depends on having the “right competence,” and the ability to cooperate with other professional groups that places the patient at the center of all activities. Quality dimensions of structural characteristics (system, documentation) and softer dimensions (dignity, relations) are important for healthcare professionals’ conceptualization of quality and should be considered in improvement activities and in daily practice. Softer dimensions of quality are challenging to measure, but further research may focus on the possibilities to transform them into measurable and relevant indicators in the healthcare.

Future researchers should include patients’ perspectives of quality in nursing homes and homecare. This might reveal differences among the perspectives of staff, their managers, and their patients [5]. It may also reveal differences between patient groups in homecare and in nursing homes. Identifying these differences and new perspectives can form a basis for future service planning, organizing, and improvement.

## Supplementary Information

**Additional file 1.** Interview guide – context individual. Interview guide used in individual interviews of managers in Norway and the Netherlands.

**Additional file 2.** Interview guide focus group employees. Interview guide used on focus. Group interviews of employees in Norwegian nursing homes and homecare services.

**Additional file 3.** Interview guide managers. Interview guide used on focus group interviews of managers in Norwegian nursing homes and homecare services.

**Additional file 4.** COREQ checklist.

## Data Availability

The data material is available on request from the corresponding author.

## Abbreviations

NH: Nursing home
HC: Homecare
