# Supplementary material for: Talking about quality: how ‘quality’ is conceptualized in nursing homes and homecare
Source: BMC Health Serv Res. 2021 Jan 30;21:104. doi: 10.1186/s12913-021-06104-0 (PMC7847031; doi:10.1186/s12913-021-06104-0)
Supplement: Supplementary file 1 — Additional file 1. Interview guide – context individual. Interview guide used in individual interviews of managers in Norway and the Netherlands. [file 12913_2021_6104_MOESM1_ESM.docx]

**Additional file 1:**

**Interview guide (individual interviews)**

**Opening questions:**

- Please, tell us about yourself
  - Age, profession, experience, how long have you been employed in your position/the work as a leader?
- How is your unit organized?
  - How do you think this organization works?
  - Is there anything you wished were different, and why?

**Introduction/transition:**

- What is your conceptualization of quality and safety?
  - How is your unit working to improve quality and safety? (for example what initiatives and efforts are implemented)
  - How do you involve employees and patients involved in this work?

**Key questions:**

- What are the key factors for your work on quality and safety?
  - At your workplace/unit/team?
  - Outside the organization?
  - (See questions under section «external factors, economy and structure” if informants need examples)
- Can you remember specific occasions were you experienced to be successful in your work to improve quality and safety? Please, tell us about it.
  - What did you do?
  - Why did it work out so well that time?
  - What were important factors for your work to turn out so well?
    - At your workplace/unit/team?
    - Outside the organization?
    - (See questions under section «external factors, economy and structure” if informants need examples)
  - Have you used these experiences to further develop your work on improving quality and safety? How?
- Can you remember specific occasions where your work with quality and safety was hampered and didn’t work out?
  - What did you do then?
  - Based on your experience, what factors hampered you work?
    - At your workplace/unit/team?
    - Outside the organization?
    - (See questions under section «external factors, economy and structure” if informants need examples)
  - Have you experienced similar occasions later? In that case, how did you managed this subsequently?
- Have you experienced one or more adverse events with large significance for your work with quality and safety? Can you please tell us about it?

**External factors, economy, and structure**

- In what way have you experienced that regulations and demands from authorities restrain or promote your work with quality and safety?
- What networks exist in the community or in your surroundings that can contribute with competence in the quality and safety improvement work?
- What influence does geographical distance (and distance to hospitals) have in your municipality for your work as a leader?
- What influence does the size of the municipality/institution have for your work on quality and safety?
- What influence does the role of political and administrative management in the municipality have on your work as a leader?
- How do the economic status influence your work with quality and safety?
- To what degree does the municipality/unit have its own strategy/plans for the quality and safety work?
- To what degree do you have access on tools, interventions and methods to work with quality and safety, and perhaps guidance from someone outside/inside the organization? (examples: report systems, internal errors, cooperation, check lists, guidance etc.).

**Closure:**

- If you were asked to make a suggestion to the politicians about what they should do in order to support your work as a leaders in improving quality and safety, what would you say?
- Is there anything else you would like to share with us to help us understand factors of importance for primary care leaders’ work on quality and safety improvement?
