# Supplementary material for: Talking about quality: how ‘quality’ is conceptualized in nursing homes and homecare
Source: BMC Health Serv Res. 2021 Jan 30;21:104. doi: 10.1186/s12913-021-06104-0 (PMC7847031; doi:10.1186/s12913-021-06104-0)
Supplement: Supplementary file 2 — Additional file 2. Interview guide focus group employees. Interview guide used on focus. Group interviews of employees in Norwegian nursing homes and homecare services. [file 12913_2021_6104_MOESM2_ESM.docx]

**Additional file 2**

**Interview guide – focus group employees**

**Introduction**

- Can you please tell about yourself; age, education, work experience, work title?

**Structure**

- What is your conceptualization of quality and safety?
- How is quality and safety work organized in this unit?
  - How is the responsibility for this work distributed? Do you know of any strategies or plans for the work?
  - How are adverse events handled?
  - Are you currently involved in any quality improvement projects? How? What kind of projects?
  - To what degree do you as employees have access to tools, measures or guides to support your quality and safety work? Can you provide examples? ,
  - Do you perceive it as beneficial to use tools/national guidelines or interventions in your daily work? Can you tell us about it?
  - What is necessary for you as employees to utilize different kind of tools in your work? (e.g., support, training)
- Do you experience having enough time specified to use evidenced based knowledge during your work?
- How do you collect and use patients and users experiences from health services in this unit?

**Culture and engagement**

- How are you working with quality and safety in this unit?
  - What challenges are you experiencing?
  - How does that affect your daily work?
- What is done in your unit to create engagement regarding quality improvement work?
- What is done in your unit to accomplish patient centered care and positive patient experiences?

**Competence**

- How does the unit facilitate competence-development among employees?
- How is it arranged for time to work with quality improvement? Is it part of the work plan?
- Who takes the initiative to implement changes related to quality and safety in the unit?
- Do you experience that changes and interventions implemented to promote quality and safety are useful in your daily work? Can you tell about it?
- How are external demands and policy guidelines adapted to fit the local needs in your unit?
- How do you experience that upper management facilitates implementation and change processes in the unit?

**Organizational politics, care coordination**

- Which networks are available for you in the quality and safety work and professional development?
- How do you experience collaboration with other professionals in the quality and safety improvement work?
- How is the collaboration with nursing homes and hospitals in this municipality facilitated?

**Psychical design/technology**

- What is your experience with data-and information systems at your unit? Do they support quality and safety improvement work? (For example reporting systems, access to data on quality, nutrition status, information from hospital, cooperation with general practitioner)
- How is the physical environment of the unit in relation to quality and safety for patients and employees? (Patients contact, reports transfer, risk of patients fall, stairs, areas outside etc.)

**External demands**

- What is your experience with local adaptions of national policy related to quality and safety improvement?
- In what way have you experienced that regulations and demands from authorities (e.g., regulatory investigations, regulations, guidelines) restrain or promote your work with quality and safety?

**Closure**

- Is there anything else you would like to share for understand factors of importance for quality and safety improvement?
