# Supplementary material for: Talking about quality: how ‘quality’ is conceptualized in nursing homes and homecare
Source: BMC Health Serv Res. 2021 Jan 30;21:104. doi: 10.1186/s12913-021-06104-0 (PMC7847031; doi:10.1186/s12913-021-06104-0)
Supplement: Supplementary file 3 — Additional file 3. Interview guide managers. Interview guide used on focus group interviews of managers in Norwegian nursing homes and homecare services. [file 12913_2021_6104_MOESM3_ESM.docx]

**Additional file 3**

**Interview guide - managers**

Background information

Age

Education / profession

Work experience

Work title

**Structure**

- What is your conceptualization of quality and safety?
- What are the key challenges in your quality and safety work?
- How is your unit working to improve quality and safety?
  - How is this work organized? (Responsibility)
  - Do you have a specific strategy and plan for quality and safety improvement work?
- Have you, as a manager, developed specific goals, strategies or plans for the quality improvement work in your unit? How does that work?
- To what extent do you have access to tools, guides, interventions, or methods to use in your quality and safety work?
  - What is your experience with using different tools in your improvement work? How often are you using them?
  - How are different kind of tools supporting your quality and safety work?
  - As a leader in a nursing home, do you miss any tools that could be useful? Can you describe it and for which purpose you would use it?
- How is the collaboration with other management levels/managers in other departments within this organization/nursing home and in the municipality?
- Do you have any experience with collecting and using patient-experiences in the quality and safety improvement work? How?
- How do you involve patients and next of kin in quality and safety improvement work?
  - Are there any specific methods or arenas for this?
- How do you handle adverse events that occur (internal reporting systems, regulatory supervision, analysis of incidents)?
  - Reporting (internally – inspectorate)
  - Information to family
  - Dealing with employees involved
  - Do you have own procedures or methods for this?
  - Which competence is there to deal with adverse events?

**Culture and engagement**

- How is a common understanding of quality and safety created? (common goals)
  - Do you experience a shared understanding and commitment towards quality and safety goals in this unit?
- What factors hamper your work on quality and safety?
- What factors facilitates your work on quality and safety?
- Can you remember specific occasions were you experienced to be successful in your work to improve quality and safety?
- Can you remember specific occasions where your work with quality and safety was hampered or didn’t work out? Why did you perceive it as such?
- How do you facilitate initiatives from employees in the quality and safety work?
- Have you experienced one or more adverse events with large influence for your work on quality and safety? Can you please tell us about it?
- How are you working to create engagement among employees in the unit regarding changes and use of evidence-based knowledge/interventions in the quality improvement work?

**Competence**

- How do you as a manager facilitate competence development among employees?
  - Which areas are of interest for the employees, and why?
- How do you map competence and competence needs among the employees? Are you doing anything specific regarding competence development in quality improvement work?
- Do employees have responsibility for following up different quality measures/goals in this unit?
- How do you work to share experiences between employees and units in quality and safety improvement work? (Also regarding adverse events)

**Organizational politics, care coordination**

- What influence does the political and administrative management in the municipality have for your work as a manager?
- How are decisions on implementation/ changes regarding quality and safety efforts made in this nursing home?
  - How do you as a manager facilitate change processes in your unit?
  - What can facilitate local adaptions to implementation in your unit?
  - How do you experience attitudes from employers when changes are necessary?
- What influence does the size of the institution/ remoteness (urban/rural) have for your work on quality and safety?
- How does the economic status influence your work with quality and safety?
- How is the cooperation/interactions with different professions in quality and safety improvement work?
- How is the interaction with hospitals in this municipality? (and between departments and other units in the municipality)

**Physical and technology environment**

- What is your experience on how data- and information’s system support quality and safety improvement? (For example reporting systems, access to data on quality, nutrition status, information from hospital, cooperation with general practitioner)
- How is the physical environment of the unit in relation to quality and safety for patients and employees? (Patients contact, reports transfer, risk of patients fall, stairs, areas outside etc.)

**External context**

- What networks exist in the municipality that can contribute with competence and support in the quality and safety improvement work?
- How do you work to balance external demands and expectancies to the employees daily work life?
- How do you as a manager work with local adaptions on national policies? How do you adjust them into the unit?
- In what way have you experienced that regulations and demands from authorities restrain or promote your work with quality and safety?
  - Do you try to influence political and national guidelines? In what way?
  - How do you establish contact with local / national media? What is the purpose of this connection?
- Have you used public reports or regulations, such as regulatory investigation reports or the regulation on leadership and quality improvement in the health and care services in your quality and safety work? How do you experience using this?

**Closure**

- Is there anything else you would like to share with us to make us understand factors of importance for quality and safety improvement?
