# Supplementary material for: Talking about quality: how ‘quality’ is conceptualized in nursing homes and homecare
Source: BMC Health Serv Res. 2021 Jan 30;21:104. doi: 10.1186/s12913-021-06104-0 (PMC7847031; doi:10.1186/s12913-021-06104-0)
Supplement: Supplementary file 4 — Additional file 4. COREQ checklist. [file 12913_2021_6104_MOESM4_ESM.docx]

**COREQ (COnsolidated criteria for REporting Qualitative research) Checklist**

A checklist of items that should be included in reports of qualitative research. You must report the page number in your manuscript where you consider each of the items listed in this checklist. If you have not included this information, either revise your manuscript accordingly before submitting or note N/A.

| **Topic** | **Item No.** | **Guide Questions/Description** | **Reported on Page No.** |
| --- | --- | --- | --- |
| **Domain 1: Research team and reflexivity** |  |  |  |
| *Personal characteristics* |  |  |  |
| Interviewer/facilitator | 1 | Which author/s conducted the interview or focus group? | p.9/10 (Data collection) |
| Credentials | 2 | What were the researcher’s credentials? E.g. PhD, MD | p.1 (Front page) |
| Occupation | 3 | What was their occupation at the time of the study? | p.1 (Front page) |
| Gender | 4 | Was the researcher male or female? | p.1 (Front page) |
| Experience and training | 5 | What experience or training did the researcher have? | p.10 (Data collection) |
| *Relationship with participants* |  |  |  |
| Relationship established | 6 | Was a relationship established prior to study commencement? | p.8 (Design, setting and sample) |
| Participant knowledge of the interviewer | 7 | What did the participants know about the researcher? e.g. personal goals, reasons for doing the research | N/A |
| Interviewer characteristics | 8 | What characteristics were reported about the inter viewer/facilitator? e.g. Bias, assumptions, reasons and interests in the research topic | p.10 (Data collection) |
| **Domain 2: Study design** |  |  |  |
| *Theoretical framework* |  |  |  |
| Methodological orientation and Theory | 9 | What methodological orientation was stated to underpin the study? e.g.  grounded theory, discourse analysis, ethnography, phenomenology, content analysis | p. 11 (Data analysis) |
| *Participant selection* |  |  |  |
| Sampling | 10 | How were participants selected? e.g. purposive, convenience, consecutive, snowball | p.8 (Design, setting and sample) |
| Method of approach | 11 | How were participants approached? e.g. face-to-face, telephone, mail, email | p.8 (Design, setting and sample) |
| Sample size | 12 | How many participants were in the study? | p.8 (Design, setting and sample) |
| Non-participation | 13 | How many people refused to participate or dropped out? Reasons? | p.8 (Design, setting and sample) |
| *Setting* |  |  |  |
| Setting of data collection | 14 | Where was the data collected? e.g. home, clinic, workplace | p.10 (Data collection) |
| Presence of nonparticipants | 15 | Was anyone else present besides the participants and researchers? | N/A |
| Description of sample | 16 | What are the important characteristics of the sample? e.g. demographic data, date | p.8 (Design, setting and sample) |
| *Data collection* |  |  |  |
| Interview guide | 17 | Were questions, prompts, guides provided by the authors? Was it pilot tested? | p.10 (Data collection) |
| Repeat interviews | 18 | Were repeat inter views carried out? If yes, how many? | N/A |
| Audio/visual recording | 19 | Did the research use audio or visual recording to collect the data? | p.11 (Data analysis) |
| Field notes | 20 | Were field notes made during and/or after the inter view or focus group? | N/A |
| Duration | 21 | What was the duration of the inter views or focus group? | p.10 (Data collection) |
| Data saturation | 22 | Was data saturation discussed? | p. 10/11 (Data collection) |
| Transcripts returned | 23 | Were transcripts returned to participants for comment and/or correction? | N/A |
| **Topic** | **Item No.** | **Guide Questions/Description** | **Reported on Page No.** |
| **Domain 3: analysis and findings** |  |  |  |
| *Data analysis* |  |  |  |
| Number of data coders | 24 | How many data coders coded the data? | p.11 (Data analysis) |
| Description of the coding tree | 25 | Did authors provide a description of the coding tree? | p.13  (Results) |
| Derivation of themes | 26 | Were themes identified in advance or derived from the data? | p.12(Data analysis) |
| Software | 27 | What software, if applicable, was used to manage the data? | N/A |
| Participant checking | 28 | Did participants provide feedback on the findings? | N/A |
| *Reporting* |  |  |  |
| Quotations presented | 29 | Were participant quotations presented to illustrate the themes/findings?  Was each quotation identified? e.g. participant number | p.14-19 (Results) |
| Data and findings consistent | 30 | Was there consistency between the data presented and the findings? | p.12(Data analysis) |
| Clarity of major themes | 31 | Were major themes clearly presented in the findings? | p.12-13(Results) |
| Clarity of minor themes | 32 | Is there a description of diverse cases or discussion of minor themes? | p.19-23 (Discussion) |

Developed from: Tong A, Sainsbury P, Craig J. Consolidated criteria for reporting qualitative research (COREQ): a 32-item checklist for interviews and focus groups. *International Journal for Quality in Health Care*. 2007. Volume 19, Number 6: pp. 349 – 357

**Once you have completed this checklist, please save a copy and upload it as part of your submission. DO NOT** **include this checklist as part of the main manuscript document. It must be uploaded as a separate file.**
